# Albuminuria and glomerular filtration rate in obese children and adolescents

**DOI:** 10.1590/2175-8239-JBN-2018-0006

**Published:** 2018-10-11

**Authors:** Luciana Satiko Sawamura, Gabrielle Gomes de Souza, Juliana Dias Gonçalves dos Santos, Fabíola Isabel Suano-Souza, Anelise Del Vecchio Gessullo, Roseli Oselka Saccardo Sarni

**Affiliations:** 1 Faculdade de Medicina do ABC Departamento de Pediatria Santo AndréSP Brasil Faculdade de Medicina do ABC, Departamento de Pediatria, Santo André, SP, Brasil.; 2 Universidade Federal de São Paulo Departamento de Pediatria São PauloSP Brasil Universidade Federal de São Paulo, Departamento de Pediatria, São Paulo, SP, Brasil.

**Keywords:** Albuminuria, Glomerular Filtration Rate, Obesity, Child, Teenager

## Abstract

**Objective:**

To describe the frequency of albuminuria in overweight and obese children and adolescents and to relate it to the severity of obesity, pubertal staging, associated morbidities and the glomerular filtration rate.

**Method:**

Cross-sectional study including 64 overweight and obese children and adolescents between 5 and 19 years of age.

**Data collected:**

weight, height, waist circumference and systemic arterial pressure.

**Laboratory tests:**

lipid profile; glycemia and insulin, used to calculate the Homeostasis Model Assessment (HOMA-IR); C-reactive protein; glutamic-pyruvic transaminase and albuminuria in an isolated urine sample (cutoff <30 mg/g). Creatinine was used to calculate the estimated glomerular filtration rate (eGFR, mL/min/1.73 m^2^).

**Results:**

The mean age was 11.6 ± 3.4 years, 32 (50%) and 29 (45.3%) were male and prepubertal. Forty-six (71.9%) had severe obesity. The frequency and median (min/max) of the observed values for albuminuria (> 30 mg/g) were 14 (21.9%) and 9.4 mg/g (0.70, -300.7 mg/g). The mean eGFR was 122.9 ± 24.7 mL/min/1.73 m^2^. There was no significant correlation between body mass index, pubertal staging, insulin and HOMA-IR with albuminuria values and neither with eGFR. Children with albuminuria tended to have higher values of diastolic blood pressure (75.0 ± 12.2 vs. 68.1 ± 12.4, p = 0.071).

**Conclusion:**

Albuminuria, although frequent in children and adolescents with obesity, was not associated with other morbidities and the glomerular filtration rate in these patients.

## INTRODUCTION

Obesity in the pediatric age group has become a worldwide epidemic in recent decades.^1^ In Brazil, the prevalence of overweight obesity has quadrupled in this age group of 5 to 9 years, in the last 30 years. Among adolescents, the prevalence of overweight increased six and three times in males and females, respectively.^2^

It is known that childhood weight gain is related to a progressive decline in renal function throughout life.^3^ In adults, the presence of albuminuria in an isolated urine sample (30-300 mg/g creatinine) is associated with a greater risk of developing type 2 diabetes, hypertension and coronary disease.^4^

The prevalence of albuminuria ranges from 0.3% to 23.9% in children and is influenced by gender, ethnicity and age. Insulin resistance, which accompanies overweight, is also involved in the pathophysiology of albuminuria.^5^

Although there is no consensus regarding the definition of metabolic syndrome in the pediatric population, it is known that the greater the number of metabolic factors altered, the greater the risk of future cardiovascular problem.^6^ Albuminuria has come to be postulated as one of the components of the metabolic syndrome; however, due to conflicting results from publications, it has been removed from the most recent classification proposals.^7^

The objective of this study was to describe the frequency of albuminuria in overweight and obese children and adolescents, to determine the severity of obesity, associated morbidity, pubertal staging and glomerular filtration rate.

## METHOD

A cross-sectional study evaluated 64 overweight and obese children and adolescents of both genders, aged 5 to 19 years, in an outpatient clinic from August 2014 to May 2015. The research protocol was approved by the Ethics Committee.

Patients with genetic or hormonal obesity were excluded; also those with other associated chronic diseases, such as urinary infection and/or hematuria; who were born less with than 37 weeks of gestation (preterm), weighing less than 2500 grams and/or were small for gestational age (SGA); (losartan, captopril, thiazide diuretics, statins, corticosteroids, and metformin). Patients using drugs that could interfere with urinary protein excretion, renal function, lipid profile and glucose tolerance were taken off the study. Thus, of the 80 patients who attended the outpatient clinic in the period, 64 (80%) were included.

In order to collect data, those responsible for children and adolescents answered a standardized questionnaire containing information on: obesity and its morbidities, socioeconomic status, personal history and family history of cardiovascular risk.

The evaluation of pubertal staging was performed by a physician according to the criteria proposed by Tanner & Marshall,^8^ taking into account the development of breasts for girls and testicles for boys.

Weight and height measures in the form of the Z score for body mass index (ZBMI) and height-for-age (ZHA) z scores, calculated with the support of the WHO ANTHRO 3.2.2 software of the World Health Organization (WHO), were used for anthropometric classification. Overweight, obesity and severe obesity were considered when +1 <ZBMI ≤ +2, +2 <ZBMI ≤ + 3 and ZBMI> +3; respectively.^9^

Abdominal circumference was measured at the midpoint between the last fixed rib and the superior border of the iliac crest. The waist-to-height ratio (WHR) above 0.5 was classified as increased, characterizing abdominal obesity.^10^

Systemic blood pressure (BP) was measured at the time of the interview, according to the Task Force's recommendation.^11^ PA values ​​were classified according to gender, age (younger and older than 13 years) and height percentile in normal BP, high BP, arterial hypertension (AH) stage 1 and AH stage 2.^11^

All measurements of BP, weight and circumference were performed by trained personnel, using calibrated equipment and periodically reviewed.

A 10 mL sample of peripheral blood was obtained after 12 hours of fasting to determine total cholesterol (TC), LDL-c, HDL-c, non-HDL cholesterol (HDL-C) and triglycerides (TG) (colorimetric method); glycemia (colorimetric method) and insulin (immunoenzymatic method), from which the Homeostasis Model Assessment (HOMA-IR) was calculated; urea and creatinine (Jaffé Modified Colorimetric Method, Roche Kit); (CRP, immunoenzymatic method) and glutamic-pyruvic transaminase (TGP, colorimetric method). The tests were performed by the Laboratory of Clinical Analyzes of the FMABC. For the lipid profile, the cut-off points recommended by the American Academy of Pediatrics were adopted; 12 for TGP were considered inadequate values above 40 U/L.

Plasma creatinine was used to calculate estimated creatinine clearance or estimated glomerular filtration rate (eGFR) according to the Schwartz equation eGFR (mL/min/1.73 m^2^) = 0.413 x stature (cm)/plasma creatinine (mg/dL))].

A separate urine sample (first morning, 20 mL) was also collected for albuminuria and creatinuria [albumin (mg)/creatinine (g)], urine I and urine culture. Albuminuria was defined as the albumin/creatinine ratio, values between: ≥30 mg/g to < 300 mg/g.^14^

For statistical analysis, we used the SPSS 24.0 software (IBM(r)). The categorical variables were presented in absolute number and percentage, compared by means of the chi-square test. The continuums were evaluated for their normality. Those following the normal distribution were presented as mean ± standard deviation and those that did not (microalbuminuria, CRP, HOMA-IR and insulin) underwent log transformation for the analyses. Student's t-test and ANOVA were used for comparison. The Pearson test was used to analyze the correlations. The significance level of 5% was adopted to rule out the null hypothesis.

## RESULTS

The general characteristics of the patients studied are presented in Table 1. The mean age was 11.6 ± 3.4 years, 32 (50%) and 29 (45.3%) were male and prepubertal, respectively.

**Table 1:** Characteristics of the study population

Variables		N = 64	%
			
Age	5 ¬ 10 years	24	37.5
	> 10 years	40	62.5
			
Gender	Males	32	50.0
	Females	32	50.0
			
Pubertal staging	Pre-pubertal	29	45.3
	Pubertal	35	54.6
			
Family history	Obesity	24	37.5
	High blood pressure	32	50.0
	Diabetes	22	34.4
	Dyslipidemia	11	17.2
	Early cardiovascular event	25	39.1
			
Blood pressure	Normal	37	57.8
	High BP	5	7.8
	Stage 1 HBP	10	15.6
	Stage 2 HBP	12	18.8
			
Lipid profile	Total cholesterol ≥ 200 mg/dL	18	28.6
	LDL-c ≥ 130 mg/dL	20	31.2
	HDL-c < 40 mg/dL	17	26.5
	Triglycerides ≥ 100 mg/dL	23	35.9
	No HDL-c ≥ 145 mg/dL	22	34.3
			
Fasting glucose	> 100 mg/dL	3	4.6
			
Glutamic-pyruvic transaminase	> 40 U/L	5	7.8
			
Albuminuria	> 30 mg/g creatinine	14	21.8

N (%)

Mean ZBMI and waist-to-height ratio were 2.9 ± 1.1 and 0.61 ± 0.08; respectively. Forty-six (71.9%) of the sixty-four patients had severe obesity (ZIMC> +3), with no difference between groups with and without albuminuria.

The most frequent obesity-related morbidities were increased waist/height ratio: 60 (93.7%), hipertrigliceridemia: 23 (35.9%), SAH: 22 (34.4%), high LDL- 31.2%); and low HDL-c: 17 (26.5%) (Table 2).

**Table 2:** Factors associated with albuminuria in obese and overweight children and adolescents

Variables		Albuminuria		
> 30 mg/g				
(n = 14)	Albuminuria			
≤ 30 mg/g				
(n = 50)	p-value			
				
Age	months	129.3±53.8	142.4±37.7	0.303^1^
				
Gender	Males	6 (42.8%)	23 (46.0%)	0.763^2^
				
Puberty	Pre-pubertal	6 (42.8%)	23 (46.0%)	0.883^2^
	Tanner 2 and 3	6 (42.8%)	18 (36.0%)	
	Tanner 4 and 5	2 (14.3%)	9 (18.0%)	
				
Body mass index	Z-score	2.8±0.9	2.9±1.2	0.662^1^
				
Waist/height	cm/cm	0.61±0.06	0.60±0.09	0.952^1^
				
Systolic BP	mmHg	116.4±16.9	109.1±14.5	0.114^1^
				
Diastolic BP	mmHg	75.0±12.2	68.1±12.4	0.071^1^
				
Total cholesterol	mg/dL	185.1±22.5	183.5±34.7	0.877^1^
				
LDL-c	mg/dL	115.4±23.2	116.4±26.8	0.907^1^
				
HDL-c	mg/dL	52.7±12.4	47.9±14.0	0.253^1^
				
Triglycerides	mg/dL	84.5±50.4	105.3±57.6	0.226^1^
				
No HDL-c	mg/dL	132.3±26.5	135.6±33.4	0.738^1^
				
Fasting glucose	mg/dL	83.5±50.3	87.1±8.9	0.178^1^
				
logHOMA-IR		1.19±0.25	1.27±0.37	0.535^1^
				
logInsulin	uU/mL	1.88±0.24	1.94±0.36	0.441^1^
				
TGP	U/L	21.1±5.4	23.8±11.3	0.404^1^
				
logPCR-us	mg/dL	1.19±0.60	1.41±0.66	0.273^1^
				
GFR	mL/min/m^2^	121.2±27.8	123.4±23.8	0.773^1^

1 Student t-test level of significance.

2 Chi-square test level of significance.

Only 3 (4.7%) patients had glycemia above 100 mg/dL and none of them were diabetic. The median (min/max) insulin and HOMA-IR were 8.5 (2.0; 37.3 uU/mL) and 1.75 (0.37, 10.02), respectively.

The frequency and median values observed for albuminuria (> 30 mg/g) were 14 (21.9%) and 9.4 mg/g (0.70, 300.7 mg/g). Only one adolescent with overweight and high BP had an albumin value in the isolated urine sample above 300 mg/g, compatible with macroalbuminuria.

**Graph 1:**
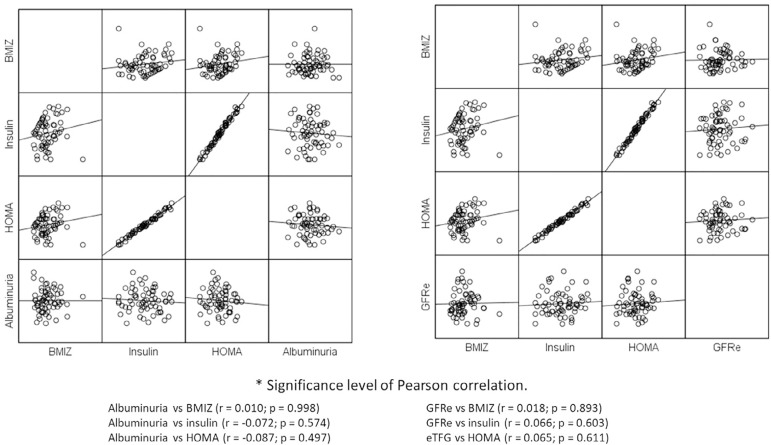
Correlation of albuminuria and glomerular filtration rate (eGFR) with z-score of body mass index (ZBMI), insulin and HOMA-IR.

The mean glomerular filtration rate (eGFR) was 122.9 ± 24.7 mL/min/1.73 m^2^ (minimum and maximum: 78.9 and 192.1 mL/min/1.73 m^2^). Four patients (6.2%) presented an eGFR lower than 90 mL/min/1.73m^2^. Of these, three were adolescents, all obese, and one had associated albuminuria. There was no significant correlation between ZBMI, insulin and HOMA-IR with albuminuria values and neither with eGFR (Graph 1).

When comparing patients with and without albuminuria, no difference was found in relation to demographic, anthropometric and laboratory variables (Table 2). Children with albuminuria showed a trend towards higher values of diastolic BP (75.0 ± 12.2 vs. 68.1 ± 12.4, p = 0.071).

**Graph 2:**
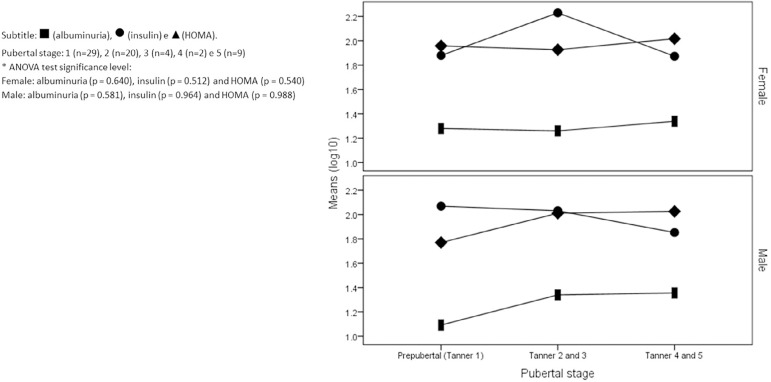
Albuminuria, insulin and HOMA-IR values according to pubertal staging in overweight and obese children and adolescents.

There was no difference in the values of albuminuria, insulin and HOMA-IR in relation to pubertal staging in boys and girls (Graph 2).

## DISCUSSION

The present study showed that one in five overweight children and adolescents had albuminuria; which was not associated with glomerular hyperfiltration, obesity severity, or overweight-related morbidities.

It is known that overweight individuals in early stages of life or who developed obesity during puberty are at higher risk of developing chronic kidney disease between 60 and 64 years.^16^ Mechanisms related to this progression are not fully elucidated, but studies suggest participation of factors such as: insulin resistance, systemic arterial hypertension, reduction of leptin concentrations, hyperaldosteronism and glomerular hyperfiltration.^17^

The presence of albuminuria is considered a good marker of renal disease and future cardiovascular risk. A recent meta-analysis, including 8 studies with adults (n = 114,105), described albuminuria as an independent predictor for cardiovascular (RR = 1.69; 95% CI 1.41-2.02) and coronary artery disease (RR = 1.41; 95% CI 1.17-1.69).^4^

In our study, the frequency of albuminuria (21.9%) was higher than that reported by other authors who evaluated overweight children and adolescents: Radhakishunna Holanda (2.7%),^18^ Lurbe in Spain (2.4%);^19^ Burgert in the USA (10.1%)^20^ and Sanad in Egypt (14.7%).^21^ Only Okpere^22^ in Nigeria (35.4%) found values higher than ours.

The severity of children and adolescents' obesity evaluated by us and the high percentage of albuminuria found are worth mentioning. This can be explained by the fact that the outpatient clinic in which the study was performed is a reference in the Greater ABC region, for more severe cases of obesity (intensity, duration and associated morbidities) in the pediatric age group.

A type of segmental and focal glomerulosclerosis is described in severely obese adults with massive proteinuria and rapid loss of renal function.^23^ Studies that sequentially evaluate albuminuria in children and adolescents with severe obesity may help in the early diagnosis of associated renal damage to increase the body mass index.

Regarding obesity-associated morbidities, insulin resistance is the one most commonly associated with albuminuria. Studies have also described the association of albuminuria with hypertriglyceridemia and systemic arterial hypertension.^24^ In turn, publications with a larger sample size, such as those performed by Radhakishun,^18^ with 408 obese patients between 2 and 18 years old, and Nguyen,^26^ with 2515 adolescents, showed divergent results. The latter, in addition to finding no association between albuminuria and cardiometabolic alterations, showed an inverse relationship between albuminuria and body mass index.

In this study, there was no association between albuminuria and the presence of morbidities associated with obesity, except for a trend towards diastolic HBP. The number of patients included and the method we used to assess glucose intolerance (HOMA alone, glucose and fasting insulin) may have influenced the outcome. A publication that more widely evaluated the glucose metabolism in a group of obese adolescents (mean age 13 years and ZBMI 2.5) found an association between the presence of albuminuria with higher glucose values at 120 minutes (in the oral tolerance test) and the area under the glycemic curve; and lower area values under the insulin curve and total insulin sensitivity (WBIS-index).^27^ There was no relationship with HOMA-IR, glycemia and fasting insulin. Although more expensive and difficult to perform, more specific laboratory methods for evaluating insulin resistance may be useful to better clarify the relationship between albuminuria and insulin resistance in obese children and adolescents.^28^^,^^29^

Obesity alone can influence eGFR. In a population study in Turkey, with a prevalence of obesity lower than ours (9.3%), they found that obese children and adolescents (5 to 18 years old) presented lower eGFR values (6.7 mL/min/1.73 m^2^) to those with normal BMI.^30^ Bonito et al.^28^ did not find any association between eGFR and BMI. However, risk factors for cardiovascular diseases, such as elevated triglycerides, fasting glucose, blood pressure, albuminuria frequency, as well as left ventricular hypertrophy, were reported more frequently in children and adolescents who had GFR values of less than 97 mL/min/1.73 m^2^ or higher than 120 mL/min/1.73 m^2^.

The natural history of renal disease can be counted through the eGFR; and values <90 mL/min/1.73 m^2^ are considered inadequate. In our study, four patients had values considered inadequate, and it was not possible to identify any common features that could justify this finding. On the other hand, hyperfiltration is also an important mechanism of kidney damage associated with obesity; however, there is no defined cut-off point for this age group.^13^

The inclusion of patients with severe obesity and the high frequency of associated morbidities may be considered strengths of this study. It should be noted that individuals with low birth weight were not included, and that the evaluation of pubertal staging was performed by pediatricians.

Some limitations of the study may be considered as the absence of a control group for comparison of albuminuria and eGFR values in a similar population without obesity. We also included patients at different stages of obesity treatment, and the limitation of the markers used to assess glucose metabolism.

It is possible to conclude that in our study albuminuria, which was frequent in obese children and adolescents, cannot be considered a marker of cardiovascular risk nor of renal injury, since it was not associated with the severity of obesity, with cardiometabolic risk factors and neither with the glomerular filtration rate.

In our study, albuminuria was frequent in obese children and adolescents. However, at this time, albuminuria was not associated with the severity of obesity, with classic cardiometabolic risk factors or with glomerular filtration rate. We believe that it is important to follow these patients, considering that factors such as age, puberty and worsening obesity may modify these findings.
